# The accuracy of the Ishii score chart in predicting sarcopenia in the elderly community in Chengdu

**DOI:** 10.1186/s12877-021-02244-4

**Published:** 2021-05-08

**Authors:** Xiaoyan Chen, Lisha Hou, Ying Zhang, Shuyue Luo, Birong Dong

**Affiliations:** 1 Zigong Mental Health Center, Zigong, Sichuan China; 2 National Clinical Research Center for Geriatrics, West China Hospital, Sichuan University, No. 37, Guo Xue Xiang Renmin Nan Lu Chengdu, Sichuan, China

**Keywords:** Sarcopenia, Elderly, Community, AWGS

## Abstract

**Background:**

Sarcopenia is a disorder associated with age that reduces the mass of skeletal muscles, the strength of muscles, and/or physical activity. It increases the risk of fall incidence which can result in fractures, hospitalizations, limited movement, and considerably decreased quality of life. Hence, it is needed to explore candidate screening tools to evaluate sarcopenia in the initial phases. The reported studies have been revealed that the sensitivity and specificity of the Ishii score chart are higher. However, the Ishii score chart is principally based on the European Working Group on Sarcopenia in Older People (EWGSOP) consensus. Recently, the Asian Working Group for Sarcopenia (AWGS) 2019 consensus has updated its diagnostic criteria for sarcopenia,which was previously similar to the EWGSOP. Hence, it is necessary to determine whether the Ishii score chart is appropriate for use among the elderly population in China. The current study aimed to validate the precision of the Ishii score chart, within the Chinese old aged community to establish an effective model for the evaluation of sarcopenia.

**Methods:**

The AWGS2019 sarcopenia diagnostic criteria were used as a standard, and among the elderly community, the accuracy of the Ishii score chart was determined by using indicators, including specificity, sensitivity, negative and positive predictive values, negative and positive likelihood ratios, Youden index, and receiver operating characteristic (ROC) curve.

**Results:**

In the elderly Chengdu community, the prevalence rate of sarcopenia was 18.38 %, 19.91 % for males and 16.91 % for females. The Ishii score chart predicts sarcopenia at an AUC value of 0.84 with 95 % confidence interval (CI), ranging between 0.80 and 0.89 for females, and at an AUC value of 0.81 with 95 % CI, ranging between 0.75 and 0.86 for males.According to the original cut-off, which was set at 120 points for females, the corresponding sensitivity was 46.91 % and the specificity was 93.22 %. The 105 cut-off points (original) set for males revealed a corresponding sensitivity of 64.94 % and the specificity of 85.46 %. However, the original cut-off value exhibited low sensitivity, hence, we selected a new cut-off value. With the new cut-off value, the sensitivity, specificity, positive and negative predictive values for sarcopenia were 75.31 %, 79.9 %, 43 %, and 94 % for females, and 70.65 %, 81.35 %, 49 %, and 92 % for males, respectively.

**Conclusions:**

The Ishii score chart was used for the prediction of sarcopenia in the old-age people of the Chengdu community and the obtained results showed a high value of predictability. Hence, more than 95 and 102 points were suggested for males and females, accordingly which can set to be the diagnostic cut-off values for the prediction of sarcopenia.

## Background

Sarcopenia is an age-associated disorder characterized by weakness of skeletal muscle strength and gradual loss of muscle mass, which may lead to the inability of physical performance [1]. This disorder badly affects the quality of life in several ways including high chances of fractures due to falling, the burden of their body weight, and subsequently a loss of mobility [2]. The underlined disorder, in the beginning, has no obvious symptoms. However, after a severe disability or fall, it becomes appear [3]. In view of these facts, it is necessary to develop significant screening strategies that can reveal the disease in the initial phases and help the elder patients within the community. At present, for sarcopenia, the frequently used screening tools are the SARC-F scale (muscle strength, assisted walking, ability to rise from chair, ability to climb stairs, and incidence of falls) [4], calf circumference (CC) [5], and the Ishii score chart [6], etc. An ideal screening model should define a reasonable cut-off point, be effective, reliable, convenient, economical, and have reasonable accuracy, sensitivity, and specificity [7]. In multiple countries, SARC-F is considered to be the most significant and commonly used tool for the screening purpose of sarcopenia. SARC-F has four subjective questions and one objective question on the screening questionnaire, the results of which may be affected by an elderly individual’s life attitude and psychology [8]. In this view, the specificity of SARC-F remains higher, and the sensitivity is lower [9, 10]. In 2019, CC was recommended by AWGS as an effective tool for the screening of sarcopenia [1]. The underlined recommendations were made on the basis of Japanese research, reported by Kawakami R et al. where calf circumference measurement was compared with dual x-ray absorptiometry (DXA) to predict the onset of sarcopenia as being 34 cm and below in males (sensitivity 88 %, specificity 91 %) and 33 cm and below in females (sensitivity 76 %, specificity 73 %) [11]. Conversely, in another study, Yves Rolland et al. retrospectively analyzed 1458 French females over the age of 70 with no history of hip fracture and found that the calf circumference was associated with skeletal muscle mass, but not sarcopenia (defined by DXA) [12]. In this study, we compared the calf circumference measurement with appendicular skeletal muscle mass (ASM) estimated by bioelectrical impedance analysis (BIA), and predicted the sarcopenia onset to be 34 cm and below in males (sensitivity 80.5 %, specificity 55.4 %) and 33 cm and below in females (sensitivity 70.9 %, specificity 77.8 %). The significance of calf circumference in sarcopenia screening is controversial. On the basis of EWGSOP consensus, the Ishii score chart was initially constructed by Ishii and his colleagues. This screening tool used inputs like age (year), grip strength (kg), and CC ( cm) as objective signs that were put into a mathematical calculation to infer the incidence of sarcopenia. The male score, for example, was calculated as follows: 0.62 ×(age-64) -3.09 ×(grip strength − 50) -4.64 ×(CC-42). Moreover, according to Ishii et al. ≥105 points was the diagnostic cut-off for sarcopenia in males [6]. Based on this, at a cut-off value of 105 in males, the sensitivity, specificity, positive, and negative predictive values for sarcopenia were 84.9 %, 88.2 %, 54.4 %, and 97.5 %. Meanwhile, the female score was calculated as follows: 0.8 ×(age-64)- 5.09 ×(grip strength − 34)- 3.28 ×(CC-42). Moreover, Ishii et al. recommended ≥ 120 points to be the diagnostic cut-off for sarcopenia in females [6]. Based on this, at a cut-off value of 120 in females, the sensitivity, specificity, positive, and negative predictive values for sarcopenia were 75.5 %, 92.0 %, 72.8 %, and 93 % respectively. More recently, Li Min [13] et al. employed the AWGS2014 consensus as the diagnostic criteria for sarcopenia to verify the accuracy of the Ishii score chart in predicting sarcopenia. They found that the sensitivity and specificity of males were 90.9 and 70.4 %, respectively. In addition, the sensitivity and specificity of females were 82.4 and 70 %, respectively. Therefore, the Ishii score chart predicts sarcopenia with high sensitivity and specificity.

Since the AWGS2019 consensus updated its prior diagnostic measures, whether the Ishii score chart is considerable for the old age Chinese population or needs further verification.This study, therefore, intends to validate the accuracy of the Ishii score chart in screening sarcopenia among the elderly Chinese population in an effort to establish a reliable and efficient model for sarcopenia screening.

## Method

### Participants

In 2014, a total of 941 elderly people with ages ranging from 60 to 92 were chosen from the Yulin, Jumper Tower, and Grout Street communities. These peoples (recruited for the current study) were living there for more than a year. They were informed by posting advertisements in the local community and by word-of-mouth by family doctors in WeChat groups. Prior to study participation, subjects signed informed consent (the participants who were unable to write issued an authorization letter to their legal guardians by providing a thumb print and the authorization letter were also signed by the legal guardians. After being authorized, the legal guardians proceeded to provide written consent on behalf of the participants and the participants then assented by providing a thumb print), and their approval was provided by the Institutional Review Committee/ Independent Ethics Committee (IRB/ IEC). Herein, the elder people with severe disorder or cancer at an advanced stage, surgical injuries to the leg/foot or hand/wrist in the last 90 days, any kind of physical disability, electronic devices, orthopedic metal implants, and those using diuretics (except for the ones who used diuretics against hypertension and the stability of dose occurs for more than 21 days), or any other disease or condition which was not considered appropriate by the investigator were excluded.

### Muscle mass measurement

Muscle mass was assessed using BIA and an Inbody720 (Inbody720, Biospace China Inc.). ASM was defined as the skeletal muscle present in the arms and legs. The values of ASM were normalized by height in meter-squared for obtaining appendicular skeletal muscle mass index (ASMI) (kg/m2) [1].

### Muscle strength measurement

Handgrip strength was used for evaluating the strength of muscles by a digital grip strength dynamometer (CAMRY EH 101), followed by conducting measurements on each hand (*n* = 3), and then the maximum values were examined.

### Physical performance measurement

Physical performance was evaluated by the typical gait speed (6 m), suitable to 0.01 s. For about 5 min, participants were indorsed to warm up and 6 m walk (twice) was performed, followed by recording and analyzing the average speed.

### Other measurements

The Tsinghua Tongfang Height and Weight Tester were used to measure the height and weight of patients. This instrument is commonly used for physical examinations in clinical practices. The measurement of height was carried out in cm (accurate to 0.1 cm), followed by measuring the weight in kg (accurate to 0.01 kg) and the CC in cm (0.1 cm). The measurement of these indices was carried out two times, followed by analyzing the average values.

### Sarcopenia classification and measurement of each component of sarcopenia

In this study, the suggestions of the AWGS2019 were followed [1]. A low muscle mass and low strength of the muscle or low physical activities are needed by proposed diagnostic criteria. The cut-off value of ASMI was 7.0 kg/m^2^ and 5.7 kg/m^2^ in males and females, accordingly, followed by recording a grip strength of < 18 kg and < 28 kg for females and males, accordingly. A gait speed of < 1.0 m/s represents a low level of physical performance.

### Statistical analysis

The stratification of statistical analysis was carried out by sex. IBM SPSS 21.0 (IBM, Armonk, NY, USA) was employed for conducting statistical analysis. A two-sided p less than 0.05 was regarded as statistically considerable. In this study, Constant data was concluded as mean ± SD or median with IQR, which is dependent on the distribution of data. Categorical variables were reported as numbers (percentage). Statistical analyses between baseline characteristics were performed using the Rank-sum, Student’s t-test, and Pearson’s chi-square test. The area under the ROC curve (AUC ) was used for evaluating the precision of the Ishii score chart in expecting sarcopenia. The AUC was not affected by population prevalence and was an ideal comprehensive index used to determine the precision of the screening tool. AUC was usually between 0.5 and 1. Moreover, the closer the AUC value was to 1, the higher was the diagnostic value of the tool. To evaluate the accuracy of the original cut-off value of the Ishii score chart for sarcopenia prediction, we assessed the following variables: specificity, sensitivity, positive and negative predictive value, as well as positive and negative likelihood ratios (LR). Furthermore, to evaluate the accuracy of the updated AWGS2019 sarcopenia diagnostic criteria, we selected a new cut-off point for predicting sarcopenia, one that maximized the Youden index. Among the variables analyzed, a positive predictive value referred to the proportion of standard-diagnosed disease cases (true positives) among subjects with positive diagnostic tests. Alternately, a negative predictive value referred to the portion of the standard-diagnosed non-disease cases (true negatives), among the negative diagnostic test subjects. The LR reflected authenticity and composed of a composite index representing sensitivity and specificity. In other words, the ratio referred to the probability of getting a test result from subjects with the condition/disease to the probability of getting the test result from subjects without the condition/disease. A positive LR represented the probability of accurately determining positive diagnosis and was calculated as a multiple of the probability of wrongly judging positive. Therefore, a highly positive LR represented a high diagnostic value of the screening tool. Conversely, a negative LR referred to the probability of a false negative and was calculated as a multiple of the probability of a true negative. In this case, a smaller negative likelihood ratio represented a higher diagnostic value. Therefore, a positive and negative LR was > 10 and < 0.1, accordingly. And the possibility of diagnosis or exclusion of disease was significantly elevated. Lastly, the Youden index was calculated as the sum of the specificity and sensitivity of the diagnostic trial minus the base value of 1 (or 100 %) and represented the overall ability of the diagnostic tool to detect true positives and true negatives. The higher the Youden index value, the higher the overall ability of the screening tool to detect true patients versus non-patients.

## Result

In 2014, a total of 941 people with ages ranging from 60 to 92 were chosen from the Yulin, Jumper Tower, and Grout Street communities.The prevalence rate of sarcopenia was 18.38 %, 19.91 % for males and 16.91 % for females, the various characteristics of participants by gender are listed in Table 1.

**Table 1:** The characteristics of participants

Variable	Total (*N* = 941)	Male (*N* = 462)	Female (*N* = 479)	*P*-value
Age (years), n(%)				0.186
< 65	306 (32.52)	146 (31.60)	160(33.40)	
65–69	240 (25.50)	110 (23.81)	130 (27.14)	
70–79	344 (36.56)	181 (39.18)	163 (34.03)	
≥ 80	51 (5.42)	25 (5.41)	26 (5.43)	
Height (m)	1.58 ± 0.84	1.64 ± 0.06	1.53 ± 0.06	< 0.001
ASMI (kg/m2)	6.65 ± 1.25	6.95 ± 1.14	6.36 ± 1.27	< 0.001
Grip strength (kg)	29.15 ± 8.96	35.66 ± 7.52	22.88 ± 4.79	< 0.001
Gait speed (m/s)	1.02 ± 0.17	1.08 ± 0.20	1.02 ± 0.17	< 0.001
Calf circumference (cm)	34.75 ± 3.00	35.54 ± 2.92	33.99 ± 2.89	< 0.001
Sarcopenia, n(%)	173 (18.38)	92 (19.91)	81 (16.91)	0.234

ASMI: appendicular skeletal muscle mass index; Sarcopenia: the presence of low muscle mass plus the presence of either low muscle strength or low physical performance; Continuous data was summarized as mean ± SD, categorical data variables were reported as numbers (percentage). Student’s t-test and Pearson’s chi-square test were performed to test the difference/associations between males and females. Student’s t-test was used on categorical data and Pearson’s chi-squared test was used on continuous data

**Fig. 1:**
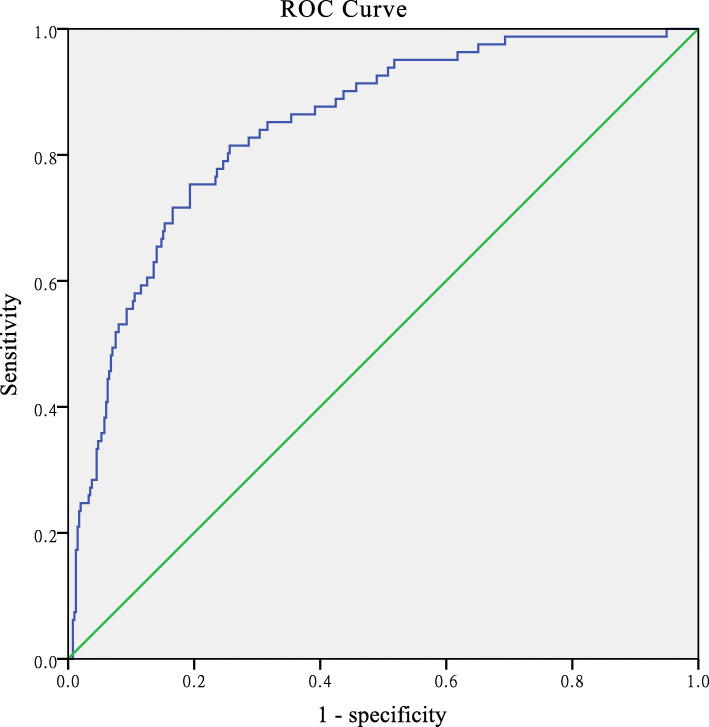
The Ishii score chart predicting sarcopenia in the female elderly community. AUC was 0.84 (95 % CI :0.80–0.89)

**Fig. 2:**
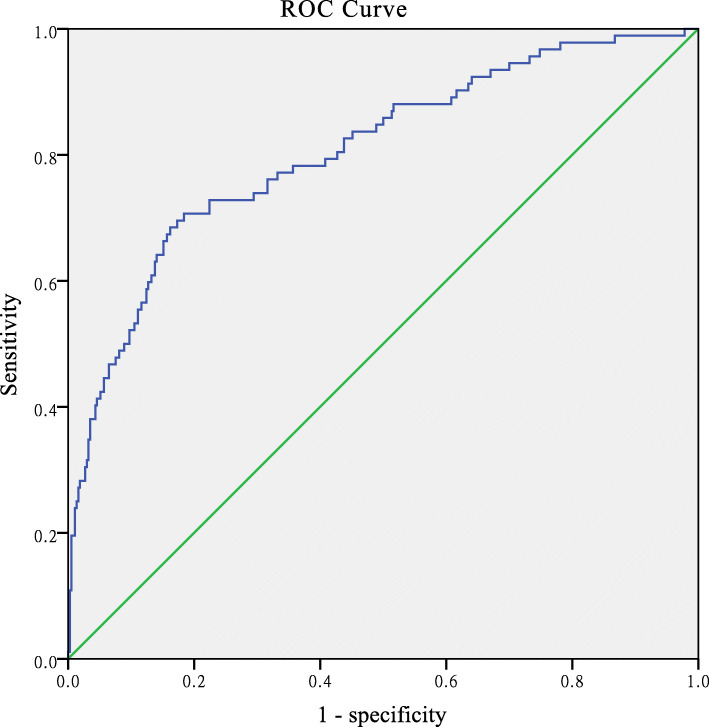
The Ishii score chart predicting sarcopenia in the male elderly community. AUC was 0.81 (95 % CI: 0.75–0.86)

Our results revealed that the Ishii score chart predicted sarcopenia in females when the AUC was 0.84 with 95 % confidence interval (CI), ranging from 0.80 to 0.89, and predicted sarcopenia in males when the AUC was 0.81 with 95 % CI, ranging from 0.75 to 0.86, as illustrated in Figs. 1 and 2.

The original cut-off point for females was 120, recommended by the Ishii score chart. The corresponding sensitivity was 46.91 % and the specificity was 93.22 %. The positive predictive value was 58 %, the negative predictive value was 90 %, the positive LR was 6.92, and the negative LR was 0.57, as shown in Table 2. The original cut-off point for males was 105, recommended by the Ishii score chart. The corresponding sensitivity, specificity, positive and negative predictive values, positive and negative LR values were 64.94 %, 85.46 %, 47 %, 92 %, 4.47, and 0.41, respectively, as shown in Table 2.

The AUC of the Ishii score chart was greater than 0.8 which leads to the identification of sarcopenia in males and females. Regarding the high predictive value, the underlined model can be used for the screening of sarcopenia in the Chinese elderly population. However, the sensitivity of the original cut-off value is low when applied to the population and required further adjustment. We, therefore, set a new cut-off value.In females, at the highest Youden index value of 102, the sensitivity and specificity of predictability were 75.31 and 79.9 %, respectively, the positive and negative predictive values were 43 and 94 %, respectively, the positive and negative LR were 3.75 and 0.31, respectively (Table 2). In males, the sensitivity and specificity of predicting sarcopenia were 70.65 and 81.35 % when the Youden index reached the highest score of 95, the positive and negative predictive, positive and negative LR values were 49 %, 92 %, 3.79, and 0.36, respectively (Table 2).

**Table 2:** The accuracy of the Ishii score chart in predicting sarcopenia

Model		Sensitivity	Specificity	Positive predictive values	Negative predictive values	Positive likelihood ratio	Negative likelihood ratio
Male							
sarcopenia	original cut-off	64.94 %	85.46 %	47 %	92 %	4.47	0.41
	new cut-off	70.65 %	81.35 %	49 %	92 %	3.79	0.36
Female							
sarcopenia	original cut-off	46.91 %	93.22 %	58 %	90 %	6.92	0.57
	new cut-off	75.31 %	79.9 %	43 %	94 %	3.75	0.31

Sarcopenia: the presence of low muscle mass plus the presence of either low muscle strength or low physical performance. The original cut-off point was recommended by the Ishii score chart: 120 for females and 105 for males; The new cut-off was the highest score of the Youden index:102 for females and 95 for males

## Discussion

In this study, the Ishii score chart was used for predicting sarcopenia (in males and females), and the obtained results revealed that the value of AUC was greater than 0.8 which shows the high value of prediction. Hence, the high predicted value of the Ishii score chart was considered as predictability of sarcopenia.

Commonly, the sensitivity and negative estimated values should be higher of the “rule-out” screening test, while the specificity and positive estimated values should be higher of the “rule-in” screening test [14]. In recent decades, screening methods, such as “rule- out” tests have been employed to determine people who are not at risk of sarcopenia. Therefore, our focus should be on the sensitivity and negative predictive values of the screening model. In the current study, the Ishii score chart was used for predicting sarcopenia, and the obtained results revealed that in males and females, the negative predictive values were 92 and 90 %, accordingly which suggested that the Ishii score chart has an elevated value of negative prediction. Moreover, in the Ishii model, the sensitivity of the original cut-off value has lower sensitivity. Generally, the sensitivity values are based on the cut-off point, which revealed that variations can occur in the values of sensitivity in accordance with the alterations in the cut-off point. Therefore, a new cut-off value was selected to increase the sensitivity of the prediction. As expected, with the new cut-off value, the Ishii scale sensitivity (in females) increased from 46.91 to 75.31 %, and the Ishii scale sensitivity (in males) in predicting sarcopenia increased from 64.94 to 70.65 %.

The current study has some limitations. First, the verification of the Ishii score chart was carried out by using selected communities in Chengdu, while in other regions, the populations were not verified. Hence, there is no clarity that whether the underlined data can be used for populations in other regions or countries. Second, all participants in this study were healthy volunteers, which may have introduced a deviation in the involved population. Therefore, further studies are needed by using a population with large sample size.

## Conclusions

The Ishii score chart was used for the prediction of sarcopenia in the old-age people of the Chengdu community and the obtained results showed a high value of predictability. Hence, more than 95 and 102 points were suggested for males and females, accordingly which can set to be the diagnostic cut-off values for the prediction of sarcopenia.

## Data Availability

The datasets generated and analyzed during the current study are not publicly available due to this is a database which has a lot of important information and we are applying some important projects based on this. But this datasets will be available 2 years later and is also available now from the corresponding author on a reasonable request.

## Abbreviations

AWGS: Asian Working Group for Sarcopenia
ASMI: Appendicular skeletal muscle mass index
ASM: Appendicular skeletal muscle mass
AUC: Area under the curve
BIA: Bioelectrical impedance analysis
CC: Calf circumference
CI: Confidence interval
DXA: Dual x-ray absorptiometry
EWGSOP: European Working Group on Sarcopenia in Older People
LR: Likelihood ratio
ROC: Receiver operating characteristic
SARC-F: Strength, Assistance with walking, Rise from a chair, climb stairs, and Falls scale
